# 

**Published:** 1911-01-15

**Authors:** 


### SUBJECT INDEX TO VOLUME LXV.

### EVENT AND COMMENT.

A Call for a New Defense Organization, 266.

American Dentistry Arraigned, 421.

Dental Educational Council of America, 214.

Exaggerated Claims for Proprietary Remedies, 265.

Get Out of the Shop, 161.

Good Teeth,—Increasing Interest, 109.

Hopeless Teeth, 110.

Metric System, 3.

"Mush Bite" 161.

National Dental Association, 213, 318, 369 and 372.

New Jersey Examiners Resignation from N. A. D. E. 56.

Ohio State Dental Society's Annual Meeting. 2.

Oral Hygiene Journal, 55.

Oral Sepsis, 577.

Prevention of Dental Caries, 317.

Reorganization of National Dental Association, 213.

Revision of Constitution of National Dental Association, 372.

Taggart Patent Agreement, 1.

The Cast Inlay Patent Situation, 525.

The Commercial in Education, 473

The Taggart Patent Suit Dismissed, 578.

Zahnarzte Haus, 162.

### FORMAL PAPERS.

A Clean Mouth Turneth Away Trouble, Wm. W. Belcher, 294.

Advantages of Nitrous Oxid-Oxygen Anesthesia, R. C. Coburn, M.D., 173.

Alveolar Abscess, Diagnosis and Treatment, A. D. Black, 448.

Artificial Feeding of Infants, 289.

A Dying Cause,—Vivisection Fallacies, J. W. Lloyd, 248.

A Method of Replacing Facings without Removal, J. W. Bryan, 394.

Ante-Natal Physiology, N. S. Hoff, 112-163.

A Plan for Raising a Fund for Unfortunate Dentists, L. G. Noel, 514.

A Review of Dr. Hunter's Address, E. C. Kirk, 597.

Asepsis in Dentistry, Joseph S. Graham, 179.

Boiled Drinking Water and Tooth Calcification, 131.

Casting Applied to Crown and Bridge Work, 228.

Casting Metals, C. N. Thompson, 16.

Dental Anatomy, N. S. Hoff, 4-58.

Dentists in the Army and Navy, Joseph D. Eby, 491.

Diagnosis and Pulp Treatment, G. L. Powers, 527.

Dr. Hunter's Address on Oral Sepsis, R. Ottolengui, 603.

Education of the Dentist, E. C. Kirk, 286.

Educating Educators, E. A. Thomas, 83.

Electricity in Dental Practice, L. E. Custer, 66.

Ethyl Chlorid Anesthesia, Open Method, Edson Abbott, 132.

Financial Investments, Henry Hall, 78.

Gold Inlay by Matrix and Solder Method, M. L. Rudolph, 435.

Immediate Root Canal Filling, P. D. Houston, 398.

Introduction of Oral Prophylaxis Treatment into Dental Practice, R. Adair, 374.

Injury to the Soft Tissues by Dentures, R. McKay, 270.

Isn't it Pathetic? J. W. Cormany, 120.

Local Anesthesia in Dental Practice, Guido Fischer, 134.

Malicious Legislation, J. W. Shedd, 558.

Medication of the Mouth, Through Dentifrices, Wallace Seccombe, 283.

Moral Aberrations due to Physical Irritants, Henry S. Upson, 277.

Mother and Child and their Relations to Oral Hygiene, W. A. White, 297.

Nitrous Oxid and Oxygen Anesthesia, E. P. Dameron, 503.

Patents and Patent Litigation, Past and Present, J. N. Crouse, 538.

Prevention of the Earlier Stages of Dental Caries, Stanley Colyer, 330.

Progress Backwards, H. L. Webster, 285.

Prophylaxis, E. C. Kirk, 127.

Prophylaxis of the First Dentition, N. S. Hoff, 320.

Prophylaxis of Childhood, N. S. Hoff, 219.

Public Service for Children, J. P. Root, 272.

Pyorrhea, Can it be Cured? A. F. James, 11.

Reorganization of the Tennessee Dental Association, A. D. Black, 486.

Rochester Outdoor School, where Delicate Children get Well and Strong, A. M. Dickinson, 300.

Silicate Cement Principles, C. M. Baldwin, 236.

The Construction of Good Vulcanite Dentures, J. H. Prothero, 560.

The Country Dentist, W. E. Green, 191.

The Demoralization of Inferior Work, Orison S. Marden, 284.

The Dentist and the Child, B. H. Johnson, 425.

The Gingival Third, J. V. Conzett, 195.

The Role of Sepsis and Antisepsis in Medicine and the Importance of Oral Sepsis as its Chief Cause, Wm. Hunter, 579.

The Significance and Diagnosis of Adenoids, Wm. D. Sumpter 475.

The X-Ray in Dentistry, J. M. King, 440.

What the Profession Owes Dr. Taggart, L. S. Tenney, 554.

### OBITUARY.

Grant Molyneux, 359.

Hugh M. Reid, 360.

Robert M. Sanger, 362.

John Q. Byram, 362.

Claude Martin, 263.

### BIBLIOGRAPHY.

Applied Anatomy and Oral Surgery, R. H. Ivy, 571.

Dental Formulary, H. Prinz, 570.

Dental Metallurgy, E. A. Smith, 357.

Essentials of Operative Dentistry, W. Clyde Davis, 357.

Hodgen's Dental Metallurgy, 571.

Johnson's Operative Dentistry, 145.

Physiology, Carl J. Wiggers, 358.

Stedman's Medical Dictionary 356.

The Business Problems of a Profession, 356.

### INDENTS.

Acolite Restorations of Badly Decayed Roots, 308.

Adrenalin for Gum Applications, 153.

Aseptic Root Filling, 253.

Bridge Swallowed, 418.

Brushing Teeth, 38.

Business Axioms, 91.

Casting with Clean Results, 417.

Casting Backings for Porcelain, 42.

Casting Saddles, 308.

Colds, the Ordinary, 89.

College's Function, 35.

Commissioned Status for Army Dental Surgeons, 412.

Conclusions, 95.

Contact Point, 295.

Death from Infected Tooth, 573.

Dental Laboratory Fire Risks, 470.

Diabetic Gingivitis, 31.

Diffusible Stimulants, 465.

Embalming Pulp Filiments, 37.

Evans Museum and Dental Institute, 520.

Fastening Tools in Wood Handles, 308.

Faulty Aluminum Castings Saved, 308.

Fletcher's Eating, 47.

Filling Cervical Cavities, 416.

Functions of the Nose, 364.

Gargling, 34.

Germicides for Pulpless Roots, 150.

Germicides Comparative Efficiency, 100.

Gum Lotions for Odontalgia, 151

Gold Inlay, 45.

Hands Kept Soft and White, 307.

Hard Plaster Models, 150.

High Pressure Anesthesia, 414.

Hunger and Thirst, 35.

Hypodermic Needles, 150.

Implantation of an Artificial Root, 414.

Incisal Cavity Preparation, 44.

Inlay Investment Mixed without Waste, 307.

Lactic Ferments in Pyorrhea Treatment, 92.

Lingual Surfaces of Vulcanite-Dentures, 102.

Lucas Splint for Retaining Lower Teeth, 366.

Make Pink Vulcanite Look Natural, 465.

Massaging, 35.

More About Water Drinking with Meals, 617.

Mouth Washes for Inflamed Gums, 152.

Mouth Washes, 205.

Modern Separation, 573.

Natural Remedies, 396.

New Local Anesthesia, 418.

Novocain Solution, 466.

Obtunding Dentine, 99.

Occlusion in Bridge Work, 413.

Plaster Cast with a Smooth Dense Surface, 466.

Plate Adhesion, 30.

Points for Busy Dentists, 97.

Poison Squad, 37.

Porcelain Crowns Cast Base, 415.

Precautions in Casting, 414.

Preventing Colds, 616.

Pressure Anesthesia for Pulp Extractions, 95.

Professional Courtesy, 39.

Prophylaxis of Enamel Fissures and Pits, 148.

Protecting Pulps under Silicate Fillings, 470.

Pulp Applications for Odontalgia, 151.

Pulp and Root Treatment, 206.

Pyorrhea with Systemic Complication, 32.

Root Impressions, 254.

Sandarac Sealing Condemned, 99.

School Children Dental Statistics, 153.

School Clinic Ideal, 147.

Saliva Ejector, 308.

Silver Nitrate Applications, 307.

Soldering, 468.

Solid Gold Dummies, 420.

Sulphurous Acid in Dentistry, 205.

Some Untoward Facts Regarding Fletcherism, 612.

Taggart Investing Technique, 41.

The Discovery of the Decade in Connection with Tuberculosis, 615.

The Open-Air School, 614.

The Prevention of Tuberculous Infection in Childhood, 615.

The Treatment of Trigeminal Neuralgia, 618.

Tin Models, 36.

To Wet the Wheel while Grinding Teeth, 465.

Treatment of Sensitive Cervical Cavities, 471.

Value of Good Teeth, 34.

Vulcanized Rubber, 36.

ANNOUNCEMENTS.

American Miller Memorial, 210.

Arkansas State Board of Examiners, 260.

Chicago Dental Society, 108, 575.

Chicago Dental Society Program, 619.

Crouse—Taggart Deal, 103.

Dental Educational Council of America, 368.

Dr. Haskell's Book, 263.

Frank Holland Banquetted, 154.

Hygiene of the Teeth, 156.

Indiana Dental Examiners, 575.

Illinois State Dental Society, 108-154.

Institute of Dental Pedagogy, 155, 576.

Indiana State Dental Association, 154.

Kentucky State Dental Association, 155, 208.

Metric System of Weights, 107.

Michigan Board of Examiners, 260, 521.

Michigan State Dental Association 107, 262,

National Dental Association, 52, 255, 310, 312.

National Dental Association Executive Council, 521.

National Association and Dr. Johnson's Paper, 522.

National, State and Local Societies, 258.

National Dental Association Clinics, 209.

New Jersey State Dental Society, 159, 261.

New Jersey Examining Board, 159.

New York Seventh and Eighth District Meeting, 367, 521.

New Orleans Odontological Society, 106.

Ohio State Dental Society, 575.

Ohio State Examiners Meeting, 208.

Oral Hygiene Conference, 313.

Oral Hygiene Lecturers, 50.

Permanent Defense Organization, 315.

Personal Mention, 261.

Protective Association Agreement with Dr. Taggart, 47.

Research Laboratory in Berlin, 209.

Southern Branch National Association, 107, 156.

South Dakota Board of Examiners 259.

St. Louis Dental College Clinic, 107, 154.

Stomatological Education, 51.

Texas State Dental Association, 160.

Virginia State Association, 208.

Washington University Dental, Clinic, 155.

Wisconsin State Dental Society, 312.

AUTOGRAPHIC.

Abbott, E., 132.

Emelin, M. J., 34.

Enomoto, Dr., 415.

Fernandez, E. M. S., 26.

Feiler, E. 471.

Ferrier, J. 131,

Finley, M. F., 106, 315.

Fischer, Guido, 134.

Fones, A. C., 148.

Gatta, J. F., 308.

Gillespie, W. C., 389.

Gillis, R. R., 97.

Graham, J. S., 179.

Greenfield, Dr. 414.

Green, W. E., 191.

Golden, Dr., 30.

Goslee, H. J., 228.

Hall, Henry, 78.

Heckard, W. H., 37.

Hoff, N. S., 4, 58, 112, 163, 219, 320.

Hunter, Wm., 579.

Houston, P. D., 398, 410.

Hudson, T. M., 446.

Hutchinson, W. G., 432.

Hutchinson, Woods, 41.

Jackman, Dr., 414.

Jackson, H. N., 39.

James, A. F., 11.

Johnson, B. H., 537.

Jones, S. R., 431, 391.

Johnson, B. H., 406, 425, 433, 480, 481.

Johnson, C. N., 44.

Kilbourn, Dr. 309.

King, J. M., 433, 440, 447, 482.

Kirk, E. C., 127, 286, 597.

Leonard, M. C., 392, 401, 407, 432, 439, 459, 484, 490, 491, 535

Lloyd, J. U., 248.

Loomis, A. G., 307.

McConachie, Dr., 365.

McKay, R., 270.

McKim, E. T., 420.

Mann, J. R., 560.

Marden, O. S., 284.

Meadows, 390, 391, 404, 444, 483.

Morgan, H. W., 386, 397, 409, 446, 491, 518.

Morrison, N. J., 428.

Neil, Dr., 518.

Newkirk, G., 47.

Noel, L. G., 391, 429, 438, 445.

O'Brien, L. A., 95.

Olds, F. L., 468.

Ottolengui, R., 603.

Patterson, J. D., 417, 472.

Pedley, J. K., 205.

Powell, G. W., 403.

Powers, G. L., 527, 537.

Prothero, J. H., 560.

Reeves, W. T., 26.

Register, H. C., 150, 465.

Rhea, C. O., 445, 482.

Rhein, M. L., 34.

Rheinstein, J. O., 418.

Rich, Celia, 396, 537.

Rich, Stanley, 388, 534, 537.

Roach, F. E., 42, 308.

Rogers, Grace, 205.

Root, J. P., 272.

Rudolph, M. L., 435, 439, 482, 513.

Samueli, Wm., 420.

Schaeffer Stuckert, 156.

Seccombe Wallace, 283.

Sevier, Dr., 511.

Simpson, O. H., 92.

Slifer, H. F., 289.

Solbrig, M. O., 150.

Sumpter, Wm. D., 475, 484.

Sutherland, R., 469.

Stetzer, J. I., 36.

Templeton, I. O., 404, 431.

Tenney, L. S., 554.

Thomas, E. A., 83.

Thompson, C. N., 16, 28.

Trueman, Wm. H. 36, 470.

Tym, W. B. 27.

Upjohn, W. H., 99.

Upson, H. S., 277.

Van Woert, Dr., 254.

Wallace, Dr., 44.

Waıren, H. 38.

Watkins, J. C., 34.

Webster, H. T., 268.

White, Goıdan, 443, 500.

White, W. A. 297.

White, C. E., 99.

Winn, J. W., 391.

Worthley, F. G., 35.

### ANNOUNCEMENTS.

Dental Protective Association Agreement with Dr. W. H. Taggart. The Dental Protective Association of the United States was organized by Dr. J. N. Crouse, of Chicago, in 1888, when it was quite common for patent claimants to collect unjust and exorbitant royalties from the members of the Dental Profession. Since this Association was organized it has succeeded in preventing patent claimants from abusing the Profession, and its strength and influence is recognized by all who are familiar with its history.

About four years ago Dr. W. H. Taggart, of Chicago, described by means of papers and clinics a new and original method of making inlay fillings. Briefly stated the method consists of making the inlay in wax and reproducing the wax inlay in gold or other metal by casting process. Since the introduction of the so-called Taggart Method, the practice of dentistry has been in a large measure revolutioinzed.

Dr. Taggart is now the owner of certain United States letters patent and applications for United States letters patent relating to the Method and Apparatus for casting inlay fillings according to this method; and he has brought suit against at least one member of the Profession and has threatened the members of the Association with litigation based upon the alleged infringement of said letters patent.

The question arose as to what action the Association would take in case any of its members were sued for alleged infringement. To engage in a long series of litigation is as unsatisfactory as it is unprofitable for all concerned. Let it be understood that the Dental Protective Association was organized to *prevent abuse* of its members, not to defraud any man of his just due. Dr. Taggart has offered to sell his machine for casting, including the permission to use the Method for $110.00, or the permission to use the Method alone for $50.00. Was this an abuse of the Dental Profession? Many dentists including Association members felt that it was not and at once purchased Taggart's machine, others felt that Dr. Taggart was asking too much since the process could be carried out successfully with a less complicated and therefore much cheaper machine.

Every dentist realized the value to him individually of the Taggart Method; and it can be said that most practitioners felt that they were in some way indebted to Dr. Taggart. How much in money value they were indebted and how the debt could be liquidated, were points which did not seem to be clear in their own minds. Therefore, the Board of Directors of the Association was urged to take the matter up with Dr. Taggart and see if amicable terms could not be made by which its members, present and prospective, could practice the Method with the machine they were now using without danger of litigation. The Board took the matter under consideration and caused an investigation to be made respecting the validity of the Letters Patent, and after being convinced that the same would probably be sustained in whole or in part if litigated, concluded that if a compromise agreement could be effected between the Association and Dr. Taggart a threefold object would be accomplished.

*1st. The members of the Association, both present and prospective, would be protected, without litigation, in case Taggart succeeded in validating his patents.*

*2nd. It would afford an opportunity of banding the Profession together into a strong and permanent Protective Association.*

*3rd. It would give Dr. Taggart, without litigation and without a hardship on any dentist, a just and reasonable compensation for his valuable invention.*

It is with a great deal of satisfation, therefore, that we are able at this time to announce to the members of the Association and to the Profession at large that an agreement in writing was entered into between the Board of Directors and Dr. Taggart, and the same was duly signed and delivered on the 5th day of December, 1910.

The terms of the agreement are such that any member of the Association in good standing can secure the present Taggart machine for the cash sum of Seventy-five ($75.00) Dollars, including the permission to use the Method, or he can obtain the permission to practice the Taggart Method alone for the life of the patents (17 years) with any machine he may now be using for the cash sum of Fifteen ($15.00) Dollars, provided the amount is paid before the entry of any decree or judgment finding Taggart's patents valid or granting damages for infringement.

The agreement further provides that any member of the Profession who joins the Association within the time above specified can also secure such permission on the same terms.

The fee for membership in the Association is Ten ($10.00) Dollars. It will be seen then that for $25.00 any member of the Profession can obtain membership in the Association and the permission to use the Taggart Method. The Board of Directors feel that this is a just and fair proposition for all concerned, and they earnestly hope that the members of the Association, both present and prospective, will realize and appreciate the delicate situation with which we were confronted and accept at once the terms of the agreement. We ask you to carefully read the accompanying amended By-Laws and trust you will immediately sign and return the same, together with your check, to the President Dr. J. N. Crouse, 2231 Prairie Ave., Chicago.

Signed

J. N. Crouse,
C. N. Johnson,
J. P. Buckley,
*Board of Directors.*

---

### ORAL HYGIENE.

Editor Dental Register.—Dear Sir: The following telegram speaks for itself:

"Albany, N. Y., December 7, 1910.

Dr. W. G. Ebersole,
Southern Hotel,
Columbus, O.

Dr. H. L. Wheeler of New York City and Dr. W. A. White of Phelps were to-day appointed by me as lecturers on Oral Hygiene for the State Department of Health.

(signed) Eugene H. Porter,

*"Commissioner of Health."*

This is the first recognition of this kind that has been given to dentistry in this country, and is the direct outcome of the Annual Conference of Health Officers of New York State which was held at Buffalo, Nov. 16th, at which time the writer addressed the Conference upon the subject of "Public Health and the Dental Profession", which was discussed by Dr. W. A. White of Phelps, N. Y., and Dr. W. W. Belcher of Rochester.

These appointments are the indirect result of the Opening of the Oral Hygiene Campaign of the National Dental Association, which was held in Cleveland on March 18th last.

Dr. W. A. White is a member of the Oral Hygiene Committee of the National Dental Association; and it is due to his activities in this direction more than to any other man that we were able to bring about these appointments. Dr. Wheeler is a member of the International Committee and has been very active for years in New York City in the Oral Hygiene field.

The acceptance of these appointments as lecturers and consultants, by these men, means that they do so at considerable sacrifice for it is not at all probable that the State will pay them for their services an amount which they could equal by confining their time to their practices.

The above telegram from Commissioner Porter was received just one year from the day the Ohio State Dental Society appropriated $500.00 for the spreading of the Oral Hygiene propaganda. The effect produced by the reading of the telegram was such that immediately following same Ohio voted to give its Committee another $500.00, and voted $100.00 to the support of the National Education and Information Bureau. This is the first contribution made by a dental organization to the support of the Oral Hygiene campaign of the National Dental Association.

W. G. Ebersole

### STOMATOLOGY.

Report of Committee on Stomatological Education. The committe on stomatological education appointed at the meeting of the American Medical Association held in St. Louis, June seventh—1910, at its first meeting held in Chicago, December second, 1910, makes the following statement.

After carefully reviewing the field with reference to matters educational in the field of medicine, one is greatly impressed with the insistence of the leaders for a better curriculum, both didactic and clinical. The inferior schools of medicine are being held up to execration. Their hour has struck. They have been "weighed in the balance and found wanting." The most pertinent reason for this is commercialism. It is a pernicious thing to place the educational and financial responsibilities in the same hands. The institution depending entirely upon the student's tuition for support is constantly exposed to great temptation. Such a college will be slow to advance the standard of preliminary education.

After a careful study of the scope and purpose of the Dental Faculties Association of American Universities, we believe that education along this line will soon reach a dignified standard which will be a credit to the nation. The whole country is to be congratulated that the University has finally addressed itself in earnest to the subject of educating the dentist.

The Dental Faculties Association of American Universities should be given all possible aid and encouragement in the work in which it is now engaged.

Signed

EUGENE S. TALBOT, D.D.S., M.D.
HERBERT A. POTTS, D.D.S., M. D.
FREDERICK B. MOOREHEAD, D. D. S., M. D.

---

### NATIONAL DENTAL ASSOCIATION.

### OFFICERS AND COMMITTEES, 1910-1911.

President: E. S. Gaylord, Connecticut; Vice-President for the East, C. S. Butler, New York; Vice-President for the West, E. R. Warner Colorado; Vice-President for the South, J. W. David, Texas; Corresponding Secretary, C. W. Rodgers, Boston, Mass.; Recording Secretary, H. C. Brown, Columbus, Ohio; Treasurer, A. R. Melendy, Knoxville, Tenn.

Executive Committee: J. D. Patterson, Chairman, Keith and Perry Bld'g, Kansas City, Mo.

First Division—Committee on Arrangements: J. D. Patterson, C. M. Works, W. G. Mason.

Second Division—Committee on Credentials and Auditing: V. H. Jackson, 240 Lenox Ave., New York City; H. B. McFadden, M. F. Finley.

Third Division—Committee on Voluntary Essays: Louis Meisburger, 85 North Pearl St., Buffalo, N. Y.; Don M. Gallie, C. J. Grieves.

Executive Council: H. J. Burkhardt, Chairman, Batavia, N. Y.; A. H. Peck, B. Holly Smith, James G. Sharp, C. L. Alexander, Edward S. Gaylord and H. C. Brown (ex-officio members).

Section 1: Stanley L. Rich, Chairman, Jackson Bld'g, Nashville, Tenn.; B. Frank Gray, Harry E. Kelsey, Secretary, 226 West Madison St., Baltimore, Md.

Section 2: L. L. Barber, Chairman, 718 Spitzer Bld'g, Toledo, Ohio; Frank I. Shaw, Seattle, Wash.; Joseph Head, Secretary, 1500 Locust St., Philadelphia, Pa.

Section 3: Wm. Carr, Chairman, 35 West 46th St., New York City; Richard Summa; Louis P. Dotterer, Secretary, Charleston, S. C.

Oral Hygiene Committee: W. G. Ebersole, Chairman, 800 Schofield Bld'g, Cleveland, Ohio; E. P. Dameron, Vice-Chairman, 58 DeMenil Bld'g, St. Louis, Mo.; Richard Grady, Secretary, Naval Academy, Annapolis, Md. J. P. Corley, Sewanee, Tenn.; W. A. White, Phelps, N. Y.; B. Holly Smith, Baltimore, Md.; H. C. Thompson, Washington, D. C.

Clinic Committee: D. O. M. LeCron, Chairman, Missouri Trust Bld'g, St. Louis, Mo.; L. A. Crumley, Vice-Chairman, 227 First Nat'l Bank Bld'g, Birmingham, Ala.; A. O. Ross, Secretary, 807 North High St., Columbus, Ohio; H. B. McFadden, Philadelphia, Pa.; H. H. Sullivan, Kansas City' Mo.; Shirley W. Bowles, Washington, D. C.; A. P. Burkhart Auburn, N. Y.; J. E. Chase, Ocala, Fla.; W. R. Clack, Mason City, Iowa; I. C. Brownlie, Denver, Colo.; Wm. Dwight Tracy, New York City; Ray D. Robinson, Los Angeles, Cal.; Charles S. Irwin, Vancouver, Wash.; F. L. Hunt, Asheville, N. C.; C. M. Barnwell, Atlanta, Ga.; Frank L. Wright, Wheeling, W. Va.; I. B. Howell, Paducah, Ky.; J. J. Sarrazin, New Orleans, La.; Richard L. Simpson, Richmond, Va.; L. B. McLaurin, Natchez, Miss.; Justin D. Towner, Memphis, Tenn.

Local Committee of Arrangements: Charles R. Butler, Honorary Chairman, Cleveland, Ohio; Geo. H. Wilson, Chairman, 701 Schofield Bld'g, Cleveland, Ohio; W. T. Jackman, Vice-Chairman, 807 Schofield Bld'g., Cleveland, Ohio; H. L. Ambler, Secretary, 428 Rose Bld'g, Cleveland, Ohio; Weston A. Price, Treasurer, 10406 Euclid Ave., Cleveland, Ohio; E. E. Belford, Cleveland, Ohio; Ira W. Brown, Cleveland, Ohio; W. G. Ebersole, Cleveland, Ohio; J. F. Stephan, Cleveland, Ohio; W. H. Whitslar, Cleveland, Ohio; A. E. Womacka.

Chairman of Sub-Committees as follôws: Publicity and Transportation: G. D. Lovett, 761 Rose Bld'g, Cleveland, Ohio; Finance: D. H. Ziegler, 716 Rose Bld'g, Cleveland, Ohio; Exhibits: S. M. Weaver, 726 Rose Bld'g, Cleveland, Ohio; Clinics: E. L. Pettibone, 5313 Franklin Ave., Cleveland, Ohio; Entertainment: A. I. Brown, 1115 New England Bld'g, Cleveland, Ohio; Hotels and Assembly Hall: H. C. Kenyon, 730 Rose Bld'g, Cleveland, Ohio; Reception: J. T. Newton, 1315 New England Bld'g, Cleveland, Ohio.

The next meeting will be held in Cleveland, July 25th to 28th, 1911.

